# Magnesium for neuroprotection in birth asphyxia

**DOI:** 10.4103/1817-1745.76094

**Published:** 2010

**Authors:** Geeta Gathwala, Atul Khera, Jagjit Singh, Bharti Balhara

**Affiliations:** Department of Pediatrics, Neonatal Services Division, Pt. B.D. Sharma Post Graduate Institute of Medical Sciences, University of Health Sciences, Rohtak, Haryana, India

**Keywords:** Asphyxia, magnesium, neuroprotection

## Abstract

**Background:**

Magnesium ion gates the N-methyl-D-aspartate (NMDA) receptor and may protect the brain from NMDA receptor-mediated asphyxial injury. The present study evaluated the neuroprotective role of magnesium in birth asphyxia.

**Material and Methods:**

Forty term neonates with severe birth asphyxia were randomized to either the study group or the control group. Neonates in the study group received magnesium sulfate in a dose of 250 mg/kg initially within half an hour of birth followed by 125 mg/kg at 24 and 48 h of birth. Cranial computed tomography (CT) scan and electroencephalography (EEG) were performed for all the babies. Denver II was used for developmental assessment at the age of 6 months.

**Results:**

Two babies in each group died of severe hypoxic ischemic encephalopathy. EEG abnormalities occurred in 43.75% of the cases in the control group compared with 31.25% in the study group. CT scan abnormalities were present in 62.5% of the control group compared with 37.5% of the cases in the study group. The Denver II assessment at 6 months revealed that there were five babies that were either abnormal or suspect in the control group compared with three in the study group.

**Conclusion:**

Magnesium is well tolerated and does appear to have beneficial effects in babies with severe asphyxia. More data is however needed and a large multicenter trial should be conducted.

## Introduction

Perinatal asphyxia is one of the major causes of neonatal mortality and long-term morbidity. Neurobiology research has extended our understanding of the mechanisms that culminate in neuronal loss after hypoxic–ischemic insult. Asphyxia leads to two types of cerebral insults: the primary neuronal injury that occurs at the time of the hypoxic–ischemic insult and the secondary neuronal injury that occurs over hours to even days following the accumulation of excessive intraneuronal calcium as a result of excitatory amino acid stimulation of the N-methyl–D-aspartate (NMDA) cell receptors.

It has been shown that NMDA receptor antagonists block calcium ion entry and preserve neuronal function and structure. MK 801, a NMDA receptor antagonist, has been shown to be neuroprotective in immature animals with asphyxia, but is too toxic to be evaluated in the human neonate.[1] Magnesium ion gates the NMDA channels in a voltage-dependent manner and may protect the brain from NMDA receptor-mediated injury.[2] In an earlier study, we have reported that a dose of 250 mg/kg and 125 mg/kg of magnesium sulfate given as an infusion is safe and well tolerated by asphyxiated neonates.[3] The present study was planned to evaluate the neuroprotective role of this dose of magnesium in birth asphyxia.

## Material and Methods

The study was performed in the Neonatal Services Division of the Department of Pediatrics, Pt. B.D. Sharma PGIMS, Rohtak. Forty term, appropriate for gestational age neonates, born in the hospital with an Apgar score of 3 or less at 1 min and 6 or less at 5 min, formed the subjects for the study. These babies were randomized to either the study group or the control group using a random number table. A preinformed consent was obtained from the parents and the study was cleared by the hospital ethics committee. Babies with congenital malformations and those whose mothers had received magnesium sulfate, pethidine, phenobarbitone or other drugs likely to depress the baby were excluded from the study.

Neonates in the study group received magnesium sulfate at a dose of 250 mg/kg initially within half an hour of birth followed by 125 mg/kg at 24 and 48 h of birth. This was given as an intravenous infusion in 5% dextrose over half an hour. Cranial computed tomography (CT) scan and electroencephalography (EEG) were performed for all the babies. The CT scan and the EEG were evaluated by examiners who were blinded to the groups to which the babies belonged. A detailed neurological examination of the neonates was carried out at the time of discharge and the babies were followed-up for neurodevelopmental assessment till the age of 6 months, when Denver II[4] was used to assess the outcome. The Unpaired “t” test and chi-square test were used for data analysis.

## Results

The mean gestational age (38.9 ± 0.4 weeks versus 38.7 ± 0.5 weeks), birth weight (2.78 ± 0.26 kg versus 2.8 ± 0.33 kg), mean cord pH (6.98 ± 0.03 versus 6.97 ± 0.02) and mean Apgar score at 1 and 5 min (1.75 ± 0.44 and 4.85 ± 1.08 versus 1.65 ± 0.5and 4.8 ± 1.19) were comparable in the control and the study groups (*P* > 0.05). Magnesium infusion was well tolerated and there were no significant alterations in heart rate, oxygen saturation, respiratory rate or mean arterial pressure following magnesium infusion either with the 250 mg/kg or the 125 mg/kg doses.

Two babies died with severe hypoxic ischemic encephalopathy in each group. An additional two babies in each group died of nosocomial sepsis. Therefore, there were 16 babies in each group that were available for follow-up assessment till the age of 6 months. Seizures occurred in 50% of the neonates in the control group compared with 35% in the study group. However, this difference was not statistically significant (*P* > 0.05). Two babies in the control group compared with one in the study group had refractory seizures. EEG abnormalities (slowing of electrical seizure activity and discontinuous pattern) occurred in 43.75% of the cases in the control group compared with 31.25% in the study group (*P* > 0.05). CT scan abnormalities (focal, multifocal or diffuse hypodensities) occurred in 62.5% of the control compared with 37.5% of the cases in the study group (*P* > 0.05) [Table 1].

**Table 1:** CT scan abnormalities

CT scan abnormality	Control group (n = 16)	Study group (n = 16)	*P*-value
Diffuse hypodensities	6 (37.5)	2 (12.5)	>0.05
Multifocal areas of hypodensity	1 (6.25)	2 (12.5)	>0.05
Focal area of hypodensity	3 (18.75)	2 (12.5)	>0.05
Total	10 (62.5)	6 (37.5)	>0.05

The mean occipitofrontal circumference at 6 months in the control group (43.11 ± 1.41 cm) was comparable to that in the study group (43.09 ± 0.86 cm). The developmental outcome using Denver II at the age of 6 months revealed that there were two babies that were abnormal in the control group versus one in the study group and there were three babies that were suspect in the control group compared with two in the study group. However, these differences between the two groups did not reach statistical significance [Table 2].

**Table 2:** Developmental outcome at 6 months: Denver II

Outcome	Control (n = 16)	Study (n = 16)	*P*-value
Normal	11 (68.7)	13 (81.2)	>0.05
Suspect	3 (18.07)	2 (12.5)	>0.05
Abnormal	2 (12.5)	1 (6.25)	>0.5

Figures in parenthesis are in percentage.

## Discussion

The study and control groups were comparable for gestational age, birth weight, Apgar scores and cord pH.

Two babies in both groups expired in the initial neonatal period as a result of asphyxia and its complications. This shows that magnesium therapy failed to prevent initial mortality due to asphyxia. However, the mortality was similar in the two groups, and no increase in mortality was seen in the group that received magnesium.

Both EEG and CT scan abnormalities occurred more frequently in the control group compared with the study group. Even though these differences did not reach statistical significance, magnesium did appear to have some beneficial effects. The follow-up assessment also revealed that more babies were either abnormal or suspect in the control group compared with the study group. Even though babies receiving magnesium appeared to have a better long-term outcome, a longer follow-up would have possibly helped to bring out the difference between the two groups better. On a 6-month follow-up, it may be possible to pick up motor abnormalities, but cognitive abnormalities that may occur as a consequence of asphyxia may be difficult to pick up at this age. A longer follow-up would have helped to pickup these cognitive abnormalities and possibly other sequelae better.

In a recently reported randomized controlled trial, magnesium sulfate was used antenatally as a neuroprotective agent in women with fetuses younger than 30 weeks and threatened preterm labor. There was reduction in the outcome of substantial gross motor dysfunction or combined outcome of death and gross motor dysfunction in the magnesium group.[5] In the index study, mortality in the magnesium group was similar to the control group, but at the 6-month follow-up, more babies were neurologically abnormal in the control group compared with the magnesium group.

In a double-blind, randomized, controlled pilot study of 22 asphyxiated full-term neonates where eight babies received magnesium sulfate, magnesium was reported to have no positive effect on the EEG patterns.[6] In the index study, EEG abnormalities occurred more frequently in the control group (44.75%) compared with the study group (31.25%). But, this difference did not reach statistical significance.

Ichibia *et al*. conducted a multicenter, randomized, controlled trial to determine whether postnatal magnesium sulfate infusion (250 mg/kg/day) for 3 days resulted in an improved outcome in babies with severe birth asphyxia.[7] Enrollment criteria included a 5-min Apgar score of 7 or less and either failure to initiate spontaneous respiration at 10 min after birth or occurrence of clinically apparent seizures within 24 h of birth. Survival with normal results on cranial CT, EEG and establishment of oral feeding by day 14 of age occurred significantly more often in the magnesium group than in the control group (12/17 versus 5/16, *P* = 0.04). In the present study too, CT scan abnormalities occurred more frequently in the control group compared with the magnesium group.

In a recent report, three doses of magnesium sulfate infusion at the 250 mg/kg per dose given daily for the first 3 days of life was shown to improve neurologic outcomes at discharge for term neonates with severe perinatal asphyxia.[8]

Experimental work supports the potential value of magnesium by several mechanisms, i.e. antiexcitotoxic (blocks the NMDA receptor), antioxidant (essential for glutathione biosyntheses), anticytokine (decreases levels of inflammatory cytokines) and antiplatelet (decreases platelet aggregation) effects.[9–15] The systemic administration of magnesium after a simulated hypoxic ischemic insult limits neurological damage in several animal models.[16–18] The limited data regarding the potential value of magnesium in the prevention of brain injury secondary to asphyxia in the human neonate are promising. More data will be of tremendous interest as prevention of mortality and morbidity associated with asphyxia will have a definite impact on neonatal survival. A larger multicenter trial could possibly better define the neuroprotective role of magnesium in asphyxia.
